# The apparency hypothesis applied to a local pharmacopoeia in the Brazilian northeast

**DOI:** 10.1186/1746-4269-10-2

**Published:** 2014-01-10

**Authors:** Alejandro Lozano, Elcida Lima Araújo, Maria Franco Trindade Medeiros, Ulysses Paulino Albuquerque

**Affiliations:** 1 Departamento de Biologia, Área de Botânica, Laboratório de Etnobiologia Aplicada e Teórica (LEA), Universidade Federal Rural de Pernambuco, Rua Dom Manoel de Medeiros s/n, Dois Irmãos, Recife 52171-030, Pernambuco, Brasil; 2 Departamento de Biologia, Área de Botânica, Laboratório de Ecologia de Ecossistemas Nordestinos, Universidade Federal Rural de Pernambuco, Rua Dom Manoel de Medeiros s/n, Dois Irmãos, Recife 52171-030, Pernambuco, Brasil; 3 Centro de Educação e Saúde, Universidade Federal de Campina Grande, Campus de Cuité, Olho d’Água da Bica, s/n, Cuité, Paraíba, Brasil

## Abstract

**Background:**

Data from an ethnobotanical study were analyzed to see if they were in agreement with the biochemical basis of the apparency hypothesis based on an analysis of a pharmacopeia in a rural community adjacent to the Araripe National Forest (Floresta Nacional do Araripe - FLONA) in northeastern Brazil. The apparency hypothesis considers two groups of plants, apparent and non-apparent, that are characterized by conspicuity for herbivores (humans) and their chemical defenses.

**Methods:**

This study involved 153 interviewees and used semi-structured interviews. The plants were grouped by habit and lignification to evaluate the behavior of these categories in terms of ethnospecies richness, use value and practical and commercial importance. Information about sites for collecting medicinal plants was also obtained. The salience of the ethnospecies was calculated. G-tests were used to test for differences in ethnospecies richness among collection sites and the Kruskal-Wallis test to identify differences in the use values of plants depending on habit and lignifications (e.g. plants were classes as woody or non-woody, the first group comprising trees, shrubs, and lignified climbers (vines) and the latter group comprising herbs and non-lignified climbers). Spearman’s correlation test was performed to relate salience to use value and these two factors with the commercial value of the plants.

**Results:**

A total of 222 medicinal plants were cited. Herbaceous and woody plants exhibited the highest ethnospecies richness, the non-woody and herbaceous plants had the most practical value (current use), and anthropogenic areas were the main sources of woody and non-woody medicinal plants; herbs and trees were equally versatile in treating diseases and did not differ with regard to use value. Trees were highlighted as the most commercially important growth habit.

**Conclusions:**

From the perspective of its biochemical fundamentals, the apparency hypothesis does not have predictive potential to explain the use value and commercial value of medicinal plants. In other hand, the herbaceous habit showed the highest ethnospecies richness in the community pharmacopeia, which is an expected prediction, corroborating the apparency hypothesis.

## Background

When applied from the ethnobotanical perspective, the apparency hypothesis, which was developed by ecologists in the 1970s (see [1,2]), treats plants as resources and humans as herbivores or consumers that require these resources [3-6]. This hypothesis considers two groups of plants, apparent and non-apparent, that are characterized by their conspicuity (apparency) for herbivores. Non-apparent plants are difficult for herbivores to find because the plants are not predictably distributed in space and time; this group includes herbs, short-cycle plants, and those at early stages of plant succession [3,5]. Apparent plants are perennial, woody plants that usually dominate forest ecosystems and are comparatively more predictable in space and time, thus being easily detected by herbivores [3,5]. In general, one could characterize non-woody plants (herbs and non-woody climbers) as non-apparent and woody ones (trees, shrubs and vines) as apparent. From the biochemical perspective of antiherbivore defense, non-apparent plants are characterized by producing qualitative defenses, such as highly bioactive, low molecular weight compounds at low concentrations (e.g., alkaloids and terpenoids), whereas apparent plants are characterized by producing digestion-reducing quantitative defenses (tannins and lignins) [3]. Apparent and non-apparent are relative terms and must be applied according to the scenario to be investigated. For example, according to one reviewer of this paper “pioneer vegetation consisting of herbs (here called non-apparent plants) is apparent immediately after human disturbance (during early successional stages)”.

The apparency hypothesis relates to plant succession in that plants characterized as non-apparent dominate the early stages of plant succession, whereas plants characterized as apparent dominate forests in later successional stages [3]. Studies conducted by different authors have shown that secondary vegetation is the main source of medicinal plants for tropical pharmacopoeias [7-9], highlighting the importance of herbs and exotic plants in some cases [10-12]. Exotic plants are related to plant succession because they usually establish themselves in disturbed habitats (secondary vegetation or cultivated areas) and most often exhibit an herbaceous habit [11]. Voeks [13] observed the importance of secondary vegetation, herbaceous habit and/or exotic species in the pharmacopoeias of different sites and concluded that tropical pharmacopoeias are primarily a product of disturbed sites that are covered by secondary vegetation.

Indices that quantify the relative value of plant species for people are required for testing the apparency hypothesis. The use value, initially presented by Phillips and Gentry [14], and relative importance, proposed by Bennett and Prance [10], are among the indices usually used for this purpose. Both of these indices are characterized by quantifying people’s knowledge of plants, but neither indicates whether the plants are actually being used [15], which limits the interpretation of their results beyond the cognitive domain. This difference between knowledge of the potential and actual use of particular plants [15,16] indicates that species with high use values do not necessarily have high practical values (current use).

Few studies have sought to ascertain whether medicinal plants belonging to secondary vegetation are actually used by the people who cite them [15]. According to the apparency hypothesis, secondary vegetation zones would be important sources of medicinal plants containing highly bioactive compounds [6,9,11,17]; therefore, one would expect these plants to be culturally important (high use value), widely used by local populations (high practical value) [15] and highlighted in the local market.

In this paper, the local pharmacopeia from a community adjacent to the Araripe National Forest (Floresta Nacional de Araripe - FLONA-Araripe) in northeastern Brazil is analyzed using habit and lignification as two means of grouping plants. The aim is to discuss the apparency hypothesis and answer the following questions: (1) Does medicinal plant richness vary between habits and between woody and non-woody plants? (2) Does contribution in terms of medicinal plant richness vary among collection sites? (3) Do use value and commercial and practical importance varies between medicinal plant habits and between woody and non-woody plants? Using the biochemical fundamentals of the apparency hypothesis, it would be expected that (1) there is a higher richness of non-woody and herbaceous medicinal species as well as (2) a higher contribution of medicinal plants from anthropogenic zones. It would also be expected that (3) non-woody and herbaceous species stand out in cognitive, practical, and commercial importance metrics.

## Methods

### Study area

The Araripe National Forest or FLONA-Araripe was established in 1946 and currently encompasses an area of 38,262.33 hectares. Most of this area is above the ‘Chapada do Araripe’ [Araripe Plateau], a formation that reaches 900 m in altitude, where the annual rainfall ranges between 671 and 2,291 mm and the average temperature is 23°C [18]. Most of the vegetation within this conservation unit is ‘Cerrado’ [wooded savannah] (48.53%), followed by ‘Cerradão’ [densely wooded savannah] (27.49%), rainforest (22.47%), and ‘Carrasco’ [xerophytic shrubby vegetation] (1.51%) [18].

The study was conducted in the rural community of Horizonte (07°29′36.9″ S, 39°22′06.02″ W) adjacent to FLONA-Araripe, located in the municipality of Jardim, Cariri region, Ceará State in northeastern Brazil. Horizonte has a registered population of 1,120 inhabitants (based on census information obtained by the community’s health workers).

Currently, there is a health-care center in Horizonte to which the population has access through consultations every Monday, when a doctor evaluates patients and provides some pharmaceutical drugs.

The residents primarily conduct subsistence agriculture. The crops typically grown include beans, cassava, and maize; only occasionally, namely when harvests are abundant, is the surplus sold. Cattle-raising activity does not generate high revenues within the region because it is conducted in the form of a small-scale production system with very low zootechnical indices [18].

The extraction of non-timber forest products is a major source of income for the area, with approximately 60% of the population over 18 claiming to do so. Resources are collected from within the FLONA and also, to a lesser extent, from private properties of the Horizonte residents. Among the species collected, ‘pequi’ (*Caryocar coriaceum* Wittm.) and the ‘fava d’anta’ tree (*Dimorphandra gardneriana* Tul.) stand out in terms of profit.

### Consent

The objectives of the project, along with the methodological procedures employed, were previously presented and discussed with the members of the community studied here, who were then asked to agree to participate and to sign a Free and Transparent Consent form (TCLE).

### Data collection

The Biodiversity Authorization and Information System (Sistema de Autorização e Informação em Biodiversidade - SISBIO) granted authorization for the present scientific activities (authorization number 27440-1), and the research ethics committee (Conselho de Ética em Pesquisa - Plataforma Brasil) provided authorization number 251.749 with CAAE 02845312.0.0000.5207 for developing this study.

Semi-structured interviews were conducted in a intensive fieldwork between March and May 2011 to collect information on medicinal plants. A total of 153 people over 18 years of age were interviewed after being randomly selected (using random sampling without replacement) from a total of 462 people in the community (according to the Department of Health census). Of the 153 interviews, 93 were with women (18 to 86 years old) and 60 were with men (18 to 80 years old). The time and place of each interview was scheduled according to each interviewee’s availability [19]. Data were checked and supplemented in the years 2012 and 2013.

The first phase of each interview was to develop a free list of the medicinal plants known to the informants and use complementary techniques, such as rereading and asking related questions (non-specifically induced) on the topic, to enrich the lists [17,19]. Once the list was completed, the interviewee was asked to mention other names that he or she knew for each of the plants previously mentioned as well as collection sites, parts used, therapeutic indications, whether the plants had any commercial value, and which parts were intended to be sold. Furthermore, the respondent was asked to identify the medicinal plants that he or she had used within the past month to obtain information on the actual use and thus the practical value of the plants; finally, the interviewee was asked to identify a resident who was knowledgeable of medicinal plants within their community.

The number of plants mentioned in the free lists was used as a quantitative criterion for choosing knowledgeable locals [19]. By combining the information in the free lists with the names of locals identified in the interviews as being knowledgeable of medicinal plants, it was possible to obtain a list of people (within the group of those randomly selected) who were best suited to participate in the study as local experts on medicinal plants. Five people were chosen as local experts: two men (63 and 48 years old) and three women (61, 44, and 31 years old), and they led individual guided tours to collect the medicinal plants mentioned in the interviews that occur within the region. A botanical sample was associated with a common name selected based on consensus among the locals experts.

During the botanical collections, the habits of the plants were identified according to the parameters proposed by Begon *et al.*[20] and adapted to the local vegetation according to Costa and Araújo [21]. Thus, woody perennials that usually had only one stem or that consistently branched at least 50 cm above the ground were classified as trees, woody perennials that were smaller and exhibited abundant branching or branches consistently below 50 cm were classified as shrubs, non-lignified plants or those only lignified at the base of the stem were classified as herbs, and woody or non-woody plants that used the structure of other plants for support were classified as climbers. Next, the plants were classes as woody or non-woody, the first group comprising trees, shrubs, and lignified climbers (vines) and the latter group comprising herbs and non-lignified climbers. In addition, the plants were also grouped according to their origin: plants that originated from outside of South America were considered to be exotic plants, and those from South America were considered natives.

The botanical collections were conducted during May and June 2011 and supplemented with collections from our team (Laboratory of Applied and Theoretical Ethnobiology) between the years 2012 and 2013. All the specimens were identified (according to APG [Angiosperm Phylogeny Group] III) and deposited at the Dárdano de Andrade and Lima Herbarium (Herbário Caririense Dárdano de Andrade e Lima - HCDAL) of the Regional University of Cariri (Universidade Regional do Cariri – URCA) in the city of Crato, Ceará. The voucher numbers run from HCDAL 6558 to 6701 and from 8104 to 8110. Duplicates were deposited at the Professor Vasconcelos Sobrinho herbarium (PEUFR) of the Federal Rural University of Pernambuco (Universidade Federal Rural de Pernambuco- UFRPE), Recife, Pernambuco.

### Data analysis

The plants were grouped by habit and lignification to test the predictions derived from the apparency hypothesis for the various aspects of the pharmacopeia analyzed.

This study used the local classification of the plants, defining as an ethnospecies each taxonomic entity considered by the interviewees. Regarding the collection sites, an ethnospecies was considered to originate from another region if 75% or more of the 153 interviewees cited it in any of the following categories: commercially traded, brought from the ‘Sertão’ (Caatinga vegetation, tropical dry forest of Northeastern Brazil surrounding the ‘Chapada do Araripe’) or brought from the ‘Cariri’ (low areas down the ‘Chapada’).

The collection sites in the FLONA region were grouped into two major types: anthropogenic areas (backyards, farms, swiddens, roadsides, paddocks, etc.) and native vegetation (vegetation present around the community on private land and vegetation from the FLONA). Each ethnospecies received a certain percentage of citations for these two types of collection sites. This percentage was used to analyze the ethnospecies richness per collection site type, determined as follows: ethnospecies with two or more informants and 75% or more citations for the site, ethnospecies with two or more informants and less than 75% citations for the site, and those with a single informant. The native vegetation was later separated into the areas outside and within the FLONA to further understand the conservation unit's role as a collection site for medicinal plants.

The plants mentioned as being used during the month preceding the interview (recently used) were analyzed to obtain information about the plants’ practical importance in relation to habit and lignification.

The use value (treated here as theoretical knowledge) was calculated according to the modified version proposed by Rossato *et al*. [22], in which VU = ∑U*i*/n, where U*i* = the number of uses mentioned by each informant for a given species and n = the total number of informants. Only the principal use for which is employed each ethnospecies is reported within this manuscript, that is, the use that received more citations from the interviewers.

The commercial value was expressed as the percentage of citations affirming sale inside and/or outside of the village. The plants considered to be commercially important were those for which more than 75% of the total interviewees stated that the plant is sold (plants with only one citation were excluded).

Using *Anthropac 4.0* software [23], the salience of the ethnospecies mentioned in the free lists of medicinal plants was calculated. Two software programs were used for statistical analyses. *BioEstat 5.0* for Windows [24] was used to conduct the G-test to test for differences in ethnospecies proportion among collection sites and the Kruskal-Wallis test to identify differences in the use values of the plants depending on habit and lignifications. Using *SPSS 17.0*, Spearman’s correlation test was performed to relate salience to use value and these two factors to the commercial value of the plants.

## Results and discussion

### Ethnospecies richness per habit

A total of 222 ethnospecies were mentioned in the free lists. Of the 222 ethnospecies, 44 were considered to come from other regions, and of the 178 ethnospecies collected from the FLONA region, 62 were herbs (36.9%), 46 were trees (27.4%), 46 were shrubs (27.4%), 13 were climbers (7.7%), and there was one parasite (0.6%) (Table 1). Ten plants were neither collected nor classified according to their habit due to difficulty in finding them in the forest or to no longer being found in backyards of the community; these plants had low numbers of citations, between one and five at the most. The herbaceous habit showed the highest ethnospecies richness in the community pharmacopeia, which is an expected result, corroborating the apparency hypothesis from the perspective of biochemical fundamentals.

**Table 1:** Medicinal plants mentioned in the free lists and collected by the locals within the FLONA region (Horizonte community, NE Brazil)

**Family/Scientific name**	**Ethnospecies**	**Principal use**	**Habit**	**Origin**	**Use value**	**Salience**	**Commercial value**	**Voucher**
**AMARANTHACEAE**								
*Alternanthera brasiliana* (L.) Kuntze	nelvagina	headache	herb	native	0.235	0.102	0.0	n.c
*Chenopodium ambrosioides* L.	mentruz	bone fracture	herb	exotic	0.497	0.137	7.7	n.c
**ANACARDIACEAE**								
*Anacardium occidentale* L.	cajueiro	healing wounds	tree	native	0.418	0.146	47.7	n.c
*Mangifera indica* L.	mangueira	breathing difficulties	tree	exotic	0.020	0.009	0.0	n.c
*Myracrodruon urundeuva* Allemão	aroeira	cutaneous illness	tree	native	0.451	0.167	10.4	6601
*Spondias purpurea* L.	ciriguela	dysentery	tree	exotic	0.170	0.071	20.0	n.c
*Spondias tuberosa* Arr.Cam.	Imbu	stomachache	tree	native	0.007	0.001	0.0	6604
*Astronium fraxinifolium* Schott.	gonçalave	flu	tree	native	0.150	0.058	20.0	6690
**ANNONACEAE**								
*Annona squamosa* L.	pinha	fever	tree	exotic	0.013	0.007	0.0	6600
*Annona coriacea* Mart.	ariticum	dysentery	shrub	native	0.150	0.073	5.9	6614
**APIACEAE**								
*Apium* sp.	milindro	high pressure	herb		0.020	0.011	0.0	6615
*Coriandrum sativum* L.	coentro	child stomachache	herb	exotic	0.013	0.003	0.0	6670
*Foeniculum vulgare* Mill.	endro	heartburn	herb	exotic	0.183	0.046	18.8	n.c
*Pimpinella anisum* L.	erva doce	general pain	herb	exotic	0.190	0.057	11.1	6606
**APOCYNACEAE**								
*Catharanthus roseus* L.	boa noite	healing wounds or scars	herb	exotic	0.007	0.004	0.0	6596
*Hancornia speciosa* Gomes	mangaba	varicose veins	tree	native	0.601	0.235	75.0	6700
*Himatanthus drasticus* (Mart.) Plumel.	janaguba	gastritis	tree	native	1.085	0.446	88.9	6660
*Secondatia floribunda* A. DC.	catuaba cipó	dilated veins	climber	native	0.026	0.010	75.0	n.c
**ARECACEAE**								
*Acrocomia aculeata* (Jacq.) Lodd. Ex Mart.	macaúba	to clean the intestine	tree	native	0.013	0.002	50.0	6590
*Attalea speciosa* Mart.	babaçu	bruise	tree	native	0.007	0.003	0.0	n.c
*Cocos nucifera* L.	côco	dehydration	tree	native	0.026	0.010	50.0	n.c
*Syagrus cearencis* Noblik	catolé	hernia	tree	native	0.013	0.010	0.0	n.c
**ASTERACEAE**								
*Acanthospermum hispidum* DC.	espinho cigano	bronchitis	herb	exotic	0.131	0.044	0.0	n.c
*Acmella oleracea* (L.) R.K. Jansen	agrião	cough	herb	native	0.046	0.009	25.0	n.c
*Agerantum conyzoides* (L.) L.	mentrasto	vaginal hemorrhage	herb	exotic	0.065	0.019	0.0	n.c
*Artemisia vulgaris* L.	anador	headache	herb	exotic	0.301	0.109	7.7	6656
*Artemisia absinthium* L.	lorma	indigestion	herb	exotic	0.183	0.085	5.6	8108
*Bidens pilosa* L.	carrapicho de agulha	woman inflammation	herb	native	0.052	0.019	16.7	6591
*Egletes viscosa* (L.) Less.	macela	digestive gases	herb	native	0.399	0.136	16.3	6603
*Helianthus annuus* L.	girassol	thrombosis	herb	exotic	0.288	0.069	13.0	6665
Indet	rosamelia	cutaneous illness	herb		0.007	0.006	0.0	6653
*Tanacetum vulgare* L.	pruma	indigestion	herb	exotic	0.085	0.034	0.0	n.c
*Vernonia condensata* Backer.	boldo de chile	heartburn	shrub	exotic	0.033	0.013	0.0	n.c
**BIGNONIACEAE**								
*Anemopaegma laeve* DC.	manacá	rheumatism	climber	native	0.013	0.005	100.0	6669
*Tabebuia* sp.	podaico	all kind of pain	tree	native	0.052	0.020	20.0	n.c
**BIXACEAE**								
*Bixa orellana* L.	urucum	hematoma	shrub	native	0.098	0.023	75.0	6698
**BORAGINACEAE**								
*Heliotropium indicum* L.	crista de galo	teething in children	herb	exotic	0.275	0.069	16.7	n.c
**BRASSICACEAE**								
*Brassica rapa* L.	mustarda	brain hemorrhage	herb	exotic	0.229	0.064	0.0	8110
**BROMELIACEAE**								
*Ananas sativus* Schult. & Schult. f.	abacaxi	flu	herb	native	0.072	0.011	14.3	6695
**BURSERACEAE**								
indet	amesca	headache	tree	native	0.007	0.002		6663
**CACTACEAE**								
*Cereus jamacaru* DC.	mandacaru	menopause	shrub	native	0.007	0.002	0.0	n.c
*Harrisia adscendens* (Gürke) Britton & Rose	rabo de raposa	menopause	shrub	native	0.020	0.008	33.3	6580
*Melocactus bahiensis* (Britton & Rose) Luetzelb	coroa de frade	magical protection	herb	native	0.007	0.001	0.0	6618
*Opuntia ficus-indica* (L.) Mill.	palma	dysentery	shrub	exotic	0.013	0.005	0.0	6696
*Pereskia grandifolia* Haw.	rosa doce	flu	shrub	native	0.020	0.009	0.0	n.c
**CANNABACEAE**								
*Cannabis sativa* L.	maconha	back pain	shrub	exotic	0.052	0.018	33.3	6574
**CAPPARACEAE**								
*Cleome spinosa* Jacq.	musambe	flu	shrub	exotic	0.039	0.016	16.7	6644
**CAPRIFOLIACEAE**								
*Sambucus australis* Cham. & Schltdl.	sabugueira	teething in children	shrub	exotic	0.026	0.010	0.0	n.c
**CARICACEAE**								
*Carica papaya* L.	mamão	constipation	shrub	exotic	0.098	0.030	9.1	6657
**CARYOCARACEAE**								
*Caryocar coriaceum* Wittm.	pequi	throat and lung pain	tree	native	0.863	0.200	98.3	6592
**CELASTRACEAE**								
*Maytenus distichophylla* Mart.	bom nome	back pain	tree	native	0.092	0.015	50.0	6613
**CONVOLVULACEAE**								
*Ipomoea batatas* (L.) Poir.	batata doce	nervousness	herb	native	0.007	0.003	100.0	6659
*Ipomoea asarifolia* (Desr.) Roem. & Schult.	salsa	itching	herb	native	0.065	0.023	50.0	6582
**COSTACEAE**								
*Costus spicatus* (jacq.) Sw.	cana de macaco	kidney stone	herb	native	0.007	0.003	0.0	6584
**CRASSULACEAE**								
*Bryophyllum pinnatum* (Lam.) Oken	malva da costa	vaginal inflammation	herb	exotic	0.680	0.270	3.1	n.c
**CUCURBITACEAE**								
*Citrullus lanatus* (Thunb.) Matsum. & Nakai	melancia	fever	herb	exotic	0.026	0.011	0.0	6583
*Cucurbita maxima* Duch. ex Lam.	abóbora	malnutrition	herb	native	0.007	0.004	0.0	n.c
*Cucurbita argyrosperma* Huber	gerimum	malnutrition	herb	exotic	0.007	0.004	0.0	n.c
*Luffa operculata* (L.) Cogn.	cabacinho	abortive	climber	native	0.013	0.008	50.0	8107
*Momordica charantia* L.	melão	itching	climber	exotic	0.020	0.007	33.3	6577
*Sechium edule* (Jacq.) Sw.	chuchu	back pain	climber	exotic	0.046	0.011	28.6	n.c
**ERYTHROXYLACEAE**								
*Erythroxylum ampliofolium* (Mart.) O.E. Schulz	catuaba	erectile dysfunction	shrub	native	0.222	0.093	80.0	6649
**EUPHORBIACEAE**								
*Croton blanchetianus* Baill.	marmeleiro	diarrhea	shrub	native	0.105	0.025	11.1	6594
*Croton campestris* A.St.-Hil.	velame	cutaneous illness	herb	native	0.490	0.175	67.3	6622
*Jatropha mollissima* (Pohl) Baill.	pinhão bravo	headache	shrub	native	0.046	0.016	0.0	6612
*Jatropha gossypiifolia* L.	pinhão roxo	neuralgia	shrub	native	0.092	0.027	12.5	6645
*Manihot esculenta* Crantz	macaxeira	malnutrition	shrub	native	0.007	0.003	0.0	8109
*Manihot esculenta* Crantz	mandioca	hematomas	shrub	native	0.033	0.010	75.0	6691
*Ricinus communis* L.	mamona	constipation	shrub	exotic	0.144	0.033	42.9	n.c
**FABACEAE**								
*Acosmium dasycarpum* (Vogel.) Yakovlev	pau pra tudo	all kind of pain	tree	native	0.111	0.058	35.7	n.c
*Amburana cearensis* (Allemão) A.C. Sm.	imburana	bronchitis	tree	native	0.314	0.076	8.3	n.c
*Bauhinia outimouta* Aubl.	mororó	teething in children	shrub	native	0.085	0.023	22.2	6647
*Bauhinia variegata* L.	pata de vaca	diabetes	tree	exotic	0.059	0.023	16.7	n.c
*Bowdichia virgilioides* Kunth	sicupira	back pain	tree	native	0.229	0.104	30.4	n.c
*Caesalpinia ferrea* C.Mart.	pau ferro	bronchitis	tree	native	0.163	0.056	41.2	n.c
*Cajanus cajan* (L.) Millsp.	andu	hoarse	shrub	exotic	0.118	0.030	8.3	n.c
*Centrosema* sp.	alcançu	bronchitis	shrub	native	0.569	0.215	70.9	6699
*Copaifera langsdorffii* Desf.	podoia	arthritis	tree	native	0.425	0.153	47.2	6661
*Crotalaria incana* L.	chucalinho	colic	shrub	native	0.013	0.004	0.0	n.c
*Dimorphandra gardneriana* Tul.	faveira	back pain	tree	native	0.059	0.093	100.0	6598
*Dioclea grandiflora* Benth.	mucunã	back pain	climber	native	0.007	0.005	100.0	n.c
*Enterolobium contortisiliquum* (Vell.) Morong	tamburí	stomachache	tree	native	0.052	0.020	0.0	n.c
*Hymenaea stignocarpa* Mart. ex. Hayne	jatobá	expectorant	tree	native	0.634	0.274	55.9	6585
*Leucaena leucocephala* (Lam.) de Wit	linhaça	high cholesterol	tree	exotic	0.105	0.024	55.6	n.c
*Mimosa pudica* L.	malicia	tooth pain	shrub	native	0.007	0.005		6689
*Parkia platycephala* Benth.	visgueiro	healing wounds	tree	native	0.020	0.008	0.0	6654
*Senna occidentalis* (L.) Link	manjerioba	abortive	shrub	native	0.072	0.029	0.0	6635
*Stryphnodendron rotundifolium* Mart.	barbatenã	healing wounds or scars	tree	native	1.144	0.451	75.7	n.c
*Vicia faba* L.	fava	carbuncle	climber	exotic	0.007	0.001	100.0	6693
*Vigna unguiculata* L., Walp.	feijão de corda	carbuncle	climber	exotic	0.026	0.002	66.7	6579
**KRAMERIACEAE**								
*Krameria tomentosa* A. St.-Hil.	carrapicho de boi	abortive	shrub	native	0.137	0.036	50.0	n.c
**LAMIACEAE**								
*Aeollanthus* sp.	hortelã	throat pain	herb	exotic				n.c
*Hyptis martiusii* Benth.	cidreira brava	flu	shrub	native	0.007	0.001	0.0	n.c
*Mentha piperita* L.	hortelã	throat pain	herb	exotic	0.915	0.397	8.2	6607
*Ocimum gratissimum* L.	alfavaca	sinusitis	herb	exotic	0.209	0.070	4.8	6605
*Ocimum americanum* L.	manjericão	inner ear pain	herb	exotic	0.144	0.044	15.4	n.c
*Ocimum basilicum* L.	manjericão	inner ear pain	herb	exotic				n.c
*Plectranthus neochilus* Schltr.	boldo	indigestion	herb	exotic	0.183	0.084	0.0	6621
*Plectranthus amboinicus* (Lour.) Spreng.	malva do reino	expectorant	herb	exotic	1.000	0.448	7.5	n.c
*Plectranthus barbatus* Andrews	sete dor	indigestion	shrub	exotic	0.170	0.072	5.3	6640
*Rhaphiodon echinus* (Nees & Mart.) Schauer	betonca	pain when urinate	herb	native	0.052	0.008	0.0	6692
*Rosmarinus officinalis* L.	alecrim	stomachache	herb	exotic	0.967	0.438	33.0	6597
**LAURACEAE**								
*Persea americana* Mill.	abacate	diuretic	tree	exotic	0.229	0.060	14.3	6595
**LYTHRACEAE**								
*Punica granatum* L.	romã	throat inflammation	shrub	exotic	0.235	0.089	51.7	6611
**MALPHIGIACEAE**								
*Byrsonima sericea* DC.	murici	hemorrhage	tree	native	0.052	0.015	16.7	n.c
*Malphigia glabra* L.	acerola	anemia	shrub	exotic	0.046	0.015	66.7	6688
*Stigmaphyllon paralias* A. Juss.	salsa parrilha	scabies	shrub	native	0.039	0.015	66.7	n.c
**MALVACEAE**								
*Abelmoschus esculentus* L. Moench	quiabo	snake bite	herb	exotic	0.007	0.006	0.0	6572
*Gossypium barbadense* L.	algodão	carbuncle	shrub	exotic	0.026	0.004	0.0	n.c
*Gossypium hirsutum* L.	algodão	carbuncle	shrub	exotic				n.c
*Sida cordifolia* L.	malva branca	teething in children	herb	native	0.216	0.068	26.3	n.c
*Waltheria indica* L.	malva	“no local use”	herb	native	0.000	0.005	100.0	n.c
**MELIACEAE**								
*Azadirachta indica* A. Juss.	neem	lice	tree	exotic	0.020	0.007	0.0	6602
**MENISPERMACEAE**								
*Cissampelos ovalifolia* DC.	orelha de onça	indigestion	herb	native	0.320	0.109	32.3	6641
								
**MUSACEAE**								
*Musa paradisiaca* L.	banana	flu	herb	exotic	0.033	0.011	0.0	6620
**MYRTACEAE**								
*Campomanesia eugenioides* (Cambess.) D.Legrand	fruta bola	malnutrition	shrub	native	0.013	0.001	50.0	n.c
*Eucalyptus citriodora* F. Muell.	eucalipto	sinusitis	tree	exotic	0.673	0.220	26.6	6576
*Eugenia uniflora* L.	pitanga	intestinal parasites	shrub	native	0.065	0.016	0.0	n.c
*Myrciaria* sp.	cambuí	the informant do not record the local use	shrub	native	0.000	0.002	0.0	6650
*Psidium guajava* L.	goiaba	healing wounds	tree	native	0.065	0.025	12.5	n.c
*Psidium guajava* L.	goiaba branca	dysentery	tree	native	0.078	0.027	12.5	n.c
*Psidium sobraleanum* Proença & Landrum	goiabinha	dysentery	shrub	native	0.065	0.033	0.0	6664
*Psidum myrsinites* DC.	araçá vermelho	diarrhea	tree	native	0.190	0.088	4.3	6619
*Syzygium cumini* (L.) Skeels	azeitona preta	diabetes	tree	exotic	0.013	0.001	0.0	6610
**NYCTAGINACEAE**								
*Boerhavia diffusa* L.	pega pinto	skin diseases in children	herb	exotic	0.059	0.014	0.0	n.c
*Mirabilis jalapa* L.	bonina	brain hemorrhage	herb	exotic	0.033	0.009	0.0	6588
**OLACACEAE**								
*Ximenia americana* L.	ameixa	healing wounds or scars	shrub	native	0.621	0.233	46.7	6571
**PASSIFLORACEAE**								
*Passiflora edulis* Sims	maracujá	insomnia	climber	native	0.085	0.026	36.4	8104
*Passiflora cincinnata* Mast.	maracujá do mato	insomnia	climber	native	0.190	0.047	5.0	6581
*Passiflora* sp.	maracujá peroba	calmative	climber	native	0.007	0.002	100.0	6648
*Turnera subulata* Sm.	xanana	renal infection	herb	native	0.092	0.030	33.3	6609
**PHYLLANTHACEAE**								
*Phyllanthus urinaria* L.	quebra pedra	kidney stone	herb	native	0.170	0.055	10.5	n.c
**PHYTHOLACCACEAE**								
*Petiveria alliacea* L.	tipí	inner ear pain	herb	native	0.052	0.010	0.0	6658
**PLANTAGINACEAE**								
*Plantago* sp.	chapeu de couro	the informant do not record the local use	herb		0.000	0.004	0.0	6638
**POACEAE**								
*Brachiaria plantaginea* (Link) Hitchc.	capim de planta	diuretic	herb	exotic	0.026	0.004	0.0	n.c
*Cymbopogon citratus* (DC) Stapf.	capim santo	calmative	herb	exotic	0.444	0.191	5.8	6628
*Cymbopogon winterianus* Jowitt ex Bor	citronela	sinusitis	herb	exotic	0.020	0.005	0.0	6586
*Saccharum officinarum* L.	cana	calmative	herb	exotic	0.131	0.039	0.0	6568
*Zea mays* L.	milho	urinary infection	herb	exotic	0.013	0.001	0.0	6646
**POLYGALACEAE**								
*Polygala paniculata* L.	caninana	rheumatism	herb	native	0.046	0.023	66.7	6642
**PROTEACEAE**								
*Roupala montana* Aubl.	congonha	liver problems	tree	native	0.124	0.039	40.0	6666
**RHAMNACEAE**								
*Ziziphus joazeiro* Mart.	juazeiro	expectorant	tree	native	0.092	0.021	20.0	n.c
**RUBIACEAE**								
*Coffea arabica* L.	café	throat pain	shrub	exotic	0.052	0.023	0.0	n.c
*Tocoyena formosa* (Cham. & Schltdl.) K.Schum.	genipapim	bone fracture	shrub	native	0.013	0.010	33.3	n.c
**RUTACEAE**								
*Citrus sinensis* (L.) Osbeck	laranja	somniferous and calmative	tree	exotic	0.340	0.107	17.1	6651
*Citrus aurantium* (L.)	laranja da terra	intestinal parasites	tree	exotic	0.020	0.009	25.0	6701
*Citrus limon* (L.) Burm. f.	limão	cold	tree	exotic	0.163	0.052	36.4	n.c
*Murraya paniculata* (L.) Jack	jasmim laranja	indigestion	shrub	exotic	0.124	0.045	0.0	n.c
*Pilocarpus microphyllus* Stapf ex Wardleworth	jaborandi	fever	shrub	native	0.046	0.019	11.1	6573
*Ruta graveolens* L.	arruda	general pain	herb	exotic	1.183	0.479	59.4	6643
**SALICACEAE**								
*Casearia javitensis* Kunth	café bravo	fatigue	shrub	native	0.007	0.002	0.0	6617
**SANTALACEAE**								
*Phoradendron mucronatum* (DC.) Krug & Urb.	estreico de passarinho	measles	parasitic	native	0.007	0.005	0.0	n.c
**SAPINDACEAE**								
*Magonia pubescens* A. St.-Hil.	tingui	healing wounds	tree	native	0.007	0.001	0.0	n.c
*Serjania* sp.	cipó de vaqueiro	hematoma	climber	native	0.007	0.004	0.0	6575
*Talisia escuelenta* A. St.-Hil.) Radlk.	pitomba	rheumatism	tree	native	0.013	0.007	0.0	6593
**SAPOTACEAE**								
*Pouteria glomerata* (Miq.) Radlk.	mamelada	cough	tree	native	0.007	0.001	0.0	6629
**SCROPHULARIACEAE**								
*Scoparia dulcis* L.	vassourinha	teething in children	herb	native	0.235	0.073	14.3	6587
**SMILACACEAE**								
*Smilax campestris* Griseb.	japecanga	cough	climber	native	0.105	0.029	62.5	6694
**SOLANACEAE**								
*Capsicum frutescens* L.	pimenta ardosa	cutaneous illness	herb	native	0.013	0.003	0.0	6570
*Solanum erianthum* D. Don	jurubeba	skin irritation	shrub	native	0.033	0.012	60.0	n.c
*Solanum* sp.	jurubeba	skin irritation	shrub					6636
*Solanum agrarium* Sendtn.	melancia da praia	menopaue	herb	native	0.052	0.019	0.0	6668
*Solanum americanum* Mill.	vamora	menopause	herb	native	0.013	0.010	0.0	6655
**URTICACEAE**								
*Pilea microphylla* (L.) Liebm.	zezinho	breathing difficulties	herb	native	0.007	0.004	0.0	6599
**VERBENACEAE**								
*Lantana camara* L.	chumbim	vaginal hemorrhage	shrub	native	0.046	0.016	25.0	6608
*Lippia gracilis* Schauer	alecrim do mato	carbuncle	shrub	native	0.026	0.016	20.0	n.c
*Lippia alba* Mill.) N.E.Br. ex Britton & P.Wilson	carmelitana	intestinal problems	shrub	native	0.020	0.012	0.0	6589
*Lippia alba* Mill.) N.E.Br. ex Britton & P.Wilson	cidreira	non appetite	shrub	native	0.569	0.244	3.3	6569
**VIOLACEAE**								
*Hybanthus calceolaria* (L.) Oken.	papaconha	teething in children	herb	native	0.346	0.101	32.3	6652
**VOCHYSIACEAE**								
*Qualea parviflora* Mart.	pau piranha	for the placenta to came out after birth	tree	native	0.052	0.027	0.0	n.c
**XANTHORRHOEACEAE**								
*Aloe vera* (L.) Burm.f.	babosa	gastritis	herb	exotic	0.412	0.099	0.0	6578
**ZINGIBERACEAE**								
*Alpinia zerumbet* (Pers.) B.L.Burtt & R.M.Sm.	colônia	low blood pressure	herb	exotic	0.098	0.036	0.0	6637

n.c = Plants not collected or not available for collection throughout the investigation period. Identified by local literature and/or material deposited in the herbaria.

In grouping the plants as woody and non-woody, we obtained 71 non-woody (42.3%) and 97 woody plants (57.7%), the latter group having the most ethnospecies, which is not in accordance with the expected results. Thus, it is clear that the type of classification used to characterize the plants defines whether the findings corroborate the predictions of the hypothesis. Because lignification is an attribute that defines apparent and non-apparent plants [3,4], the classification of plants as woody and non-woody is more suited for the apparency hypothesis; furthermore, it is a dichotomous grouping with two mutually exclusive groups. Despite this, the two classification types clearly define the groups that represent non-apparent plants (herbs and non-woody) and allow testing of the predictions made in the present study.

The dominance of herbaceous plants in terms of medicinal species richness has also been reported in pharmacopoeias from other Brazilian ecosystems, such as the Caatinga [semi-arid vegetation] [25] and Atlantic Forest [12], and also in other locations such as Southern Mexico [11]. In the study by Albuquerque *et al*. [25], woody (trees, shrubs, and sub-shrubs) and non-woody (herbaceous) plants totaled 56.6% and 43.4% of medicinal plants, respectively, proportions similar to those found in the present study, showing that woody plants exhibited the higher medicinal species richness. In contrast, in the study by Voeks [12], using either of the two types of grouping proposed in the present study, the prediction of dominance by non-woody (herbaceous) plants based on the biochemical fundamentals of the apparency hypothesis was corroborated. In that study, woody (trees and shrubs) and non-woody plants (herbs and epiphytes) totaled 35% and 56% of the medicinal plants, respectively (lianas, the remaining 9%, were not included because they are both woody and non-woody). This result indicates that non-woody medicinal plants, specifically the herbaceous ones, dominate the pharmacopeia in the Brazilian Atlantic Forest.

**Figure 1:**
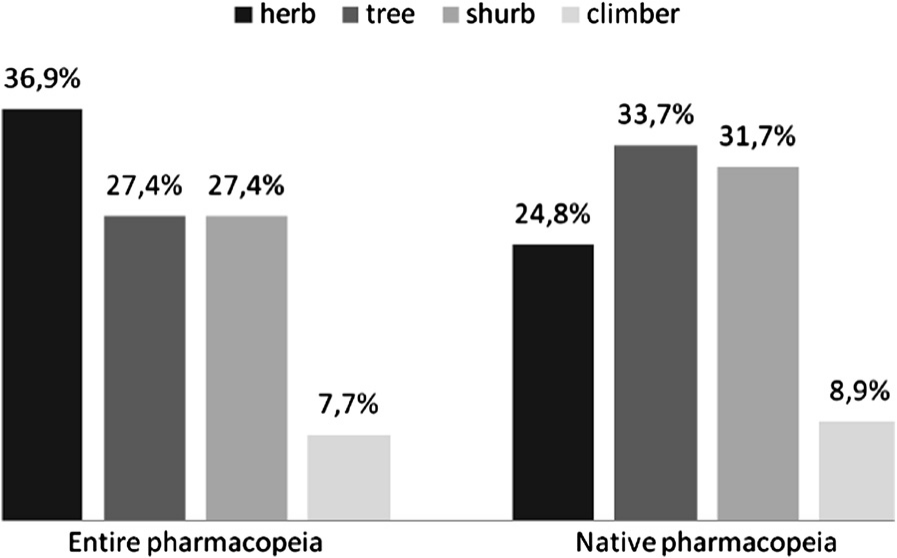
Percentage of ethnospecies per habit in the free lists (entire pharmacopeia) and after removing the exotic ethnospecies (native pharmacopoeia) in Horizonte community, NE Brazil.

A total of 64 (38.8%) exotic and 101 (61.2%) native ethnospecies were reported among the medicinal plants collected within the FLONA region (the origin could not be determined for three ethnospecies due to taxonomic difficulties). The exotic plants were divided into 34 herbs (53.1%), 14 shrubs (21.9%), 12 trees (18.7%), and four climbers (6.3%). When the exotic plants were excluded from the analysis of the native pharmacopoeia, 34 trees (33.7%), 32 shrubs (31.7%), 25 herbs (24.8%), nine climbers (8.9%), and one parasite (0.9%) were recorded, indicating that the dominance of herbaceous plants in this pharmacopeia is an effect of the exotic medicinal flora (Figure 1). With the removal of the exotic plants, the difference between the percentages of woody and non-woody plants in the pharmacopoeia increased, resulting in 30 non-woody (29.7%) and 71 woody species (70.3%). In the case of the native pharmacopoeia, the woody plants (mostly of arboreal habit) dominated, a finding that was also reported for the Caatinga [26] and does not match the predictions made based on the apparency hypothesis from the perspective of biochemical fundamentals. Thus, the influx of medicinal species from other continents than South America has an effect on the proportion of habits of the plants in the local pharmacopeia and, therefore, on interpreting this pharmacopoeia from the perspective of the apparency hypothesis.

Although outside of the scope of this study, it is important to mention that the apparency hypothesis has other approximations. Phillips and Gentry [27] proposed that, from an ecological perspective, apparent (conspicuous) plants are the more abundant ones in the vegetation and therefore subject to be experienced more frequently by human communities, which will lead to a major development of uses for those. Based on this idea, one could suppose that if a plant species is widespread and abundant in a certain environment it is apparent regardless of its habit but this assumption has not yet been tested.

### Habits of ethnospecies and their practical values

A total of 90 ethnospecies were reportedly used during the month preceding the day of the interview (i.e., were recently used). Of these ethnospecies, 11 were from other regions and 79 were collected within the FLONA region; the latter can be divided into the following groups: 37 herbs (46.8%), 21 trees (26.6%), 17 shrubs (21.5%), 3 climbers (3.8%), and one plant of indeterminate habit (one plant not collected). Again, the herbaceous habit was characteristic of most of the ethnospecies. The proportion of herbs increased in the recently used plants while the other habits decreased compared to the percentages of plants per habit in the free lists, suggesting that herbaceous plants were the habit of most practical importance.

Supporting these results, among the plants used recently, 40 were non-woody (50.6%) and 38 were woody species (48.1%), increasing the percentage of non-woody plants to the point of surpassing the percentage of woody plants when compared to the values observed in the free lists. These results, obtained via both means of grouping the plants to test the apparency hypothesis, suggest that non-woody plants, specifically those of herbaceous habit, have the most practical value. This finding strongly indicates that these plants, characterized from the biochemical perspective of the apparency hypothesis as having highly bioactive compounds content, are the most desirable for treating diseases within the Horizonte community.

The results obtained for the plants used during the month preceding the interviews are influenced by various factors that may skew our interpretation. The period of the year when the interviews were conducted corresponded to the region’s rainy season, concentrated from December to April [18], a period in which one would expect the herbaceous component of the vegetation to be fully available in the environment, and that may have led to greater use of plants with that habit. These results may also reflect the pattern of diseases experienced by Horizonte inhabitants during this period (e.g., a cold virus spread throughout the population), leading to the use of certain plants.

Exotic plants corresponded to 50% (39 ethnospecies) of the medicinal plants used during the month preceding the interview, having a higher representation than in the entire pharmacopeia (38.8%). When the exotic species were removed from the group of plants mentioned as being used within the last month, the percentage of ethnospecies per habit changed, highlighting the following distribution among the native plants used: 14 ethnospecies each corresponded to trees (35.9%) and to herbs (35.9%), followed by 9 shrubs (23%) and two climbers (5.1%). The exotic medicinal flora highlights the herbaceous habit, this time on the practical level because when the exotic plants are removed, trees and herbs formed the highest percentages of ethnospecies richness among those recently used.

### Collection sites

The results of the informant consensus associating ethnospecies to collection sites are shown in Table 2. In this analysis, the term “other regions” was considered to refer to collection sites that, although not sites in the sense of areas, are still sources of medicines for Horizonte residents; including this term allows for comparisons with this group of plants.

**Table 2:** Medicinal ethnospecies richness per collection site with two or more informants and 75% or more citations for the site, with two or more informants and less than 75% citations for the site, and with only one informant (Horizonte community, NE Brazil)

		**Collection sites**					
		**Anthropogenic areas**		**Native vegetation**		**Other regions**	
**Ethnospecies richness**		FL	RU	FL	RU	FL	RU
	≥ 75% citations	90	54	27	13	26	10
	< 75% citations	57	29	42	26	28	15
	Only one informant	26	1	8	0	18	1
	Total	173	84	77	39	72	26

The analysis was based on the free lists (FL) and lists of plants recently used (RU). Horizonte community, NE Brazil.

The ethnospecies richness cited in the free lists exhibited significant differences depending on the collection sites (X^2^ 16.40; 4 df [degrees of freedom]; p < 0.01), which corresponded to differences between anthropogenic areas and native vegetation (X^2^ = 10.39; 2 df; p < 0.01), between anthropogenic areas and other regions (X^2^ = 6.05; 2 df; p < 0.05), and between native vegetation and other regions (X^2^ = 6.50; 2 df; p < 0.05).

The anthropogenic areas differed significantly from other regions in being the site with the highest number of ethnospecies cited and were the principal source (collecting site) of medicinal plants for the Horizonte inhabitants. This result reinforces the findings of other authors that secondary vegetation or the vegetation of anthropogenic areas is the main source of medicinal plants in the pharmacopoeia of human communities in tropical forests [7,28-30].

Based on the informant consensus, 72.6% (45 ethnospecies) of all the plants, 34.8% (16) of the trees, 50% (23) of the shrubs, and 38.5% (5) of the climbers were strongly associated with collection in anthropogenic areas. When the plants were categorized as woody and non-woody, 70.4% (50) of the non-woody and 40.2% (39) of the woody plants were strongly associated with collection in anthropogenic areas. Additionally, 79.7% (51) of exotic plants were strongly associated with these areas. These results indicate a close relationship between anthropogenic areas and the collection of non-woody medicinal plants, mostly those with an herbaceous habit, as well as of exotic plants.

The greater value associated with anthropogenic areas was an expected result from the perspective of the biochemical fundamentals of the apparency hypothesis because anthropogenic areas, in our classification, are open and cultivated areas as well as areas with vegetation in the early stages of plant succession. Therefore, these regions were considered to be areas where non-apparent species dominate, a fact that was corroborated by the high incidence of reports of collection of non-woody herbaceous plants. The greater presence of woody plants, primarily shrubs, in anthropogenic areas certainly helps in differentiating these regions as the main source of medicinal plants and contributes to the higher importance attributed to these areas. Among the woody plants strongly associated with collection in anthropogenic areas, exotic species corresponded to 52.2% of the shrubs and 62.5% of the trees, indicating that the contribution of woody plants to the higher importance of anthropogenic areas as medicinal collection sites was, in more than half of the cases, due to the contribution of exotic plants.

Explanations of both an environmental and a cultural nature may contribute to understanding the role of anthropogenic areas and native vegetation in tropical pharmacopoeias. In the Brazilian semi-arid lands within the Caatinga ecosystem, native vegetation zones are the primary source of medicinal plants that are effectively used [15]. This pattern makes sense because in seasonal climates such as that of the Caatinga, where annual rainfall is irregularly distributed throughout the year and ranges between 250 and 1,200 mm, plant use is directed toward the perennial parts of woody plants (bark and roots) that are available throughout the year, in contrast to the herbaceous plants that have limited availability during the dry season [6]. Thus, there is a preference for using medicinal woody plants sought primarily in areas of native vegetation [17,31].

Explanations of a cultural nature may also contribute to understanding the dominance of herbs and anthropogenic areas in tropical forest pharmacopoeias. For example, in the Brazilian Atlantic Forest, where the annual rainfall is evenly distributed and varies between 1,800 and 2,100 mm, the limited contact that slaves historically had with the region”s primary vegetation (due to their captivity and work in cultivated areas coupled with a high rate of deforestation in this ecosystem) was a factor reported by Voeks [12] to explain why anthropogenic areas and herbaceous plants are important in the current local pharmacopeia. One must also consider the influence of other cultural archetypes that have been important in forming the local pharmacopoeia over time, of which knowledge and practices are also present in the current local pharmacopeia to varying extents [32]. In this regard, the presence of the dominant European discourse, which attempted to impose its lifestyles on the inhabitants of the “new lands”, is also noteworthy [33]. Many European medicines were incorporated into the African-Brazilian pharmacopoeia, which were either cultivated or found in local secondary vegetation [12].

However, following the line of explanations from the environmental standpoint, it is expected that in Atlantic Forest regions, due to the lack of a dry season [34], herbs and anthropogenic areas became prominent as the main sources of medicinal plants. The ‘Cerrado’ biome, with annual rainfall between 800 and 2,000 mm and a marked dry period [35], is intermediate between the Caatinga and Atlantic Forest biomes in terms of annual rainfall. The results obtained regarding the greater prominence of herbaceous plants in terms of ethnospecies richness and practical importance are similar to those found by other authors in tropical rainforests [12,29].

The native vegetation is primarily a source of medicinal woody flora. Of the 27 native plants that are strongly associated with collection in the native vegetation, 96.1% (25 ethnospecies) are native woody species. Upon separating the native vegetation between outside and inside the FLONA to evaluate the conservation unit’s role as a site for gathering medicinal plants, there were no significant differences in the ethnospecies richness of the plants that were cited as collected at these two sites (G test 5.44; 2 df; p > 0.05). This result indicates that the Horizonte residents find medicinal plants in the native vegetation that can be collected both outside and within the FLONA.

**Figure 2:**
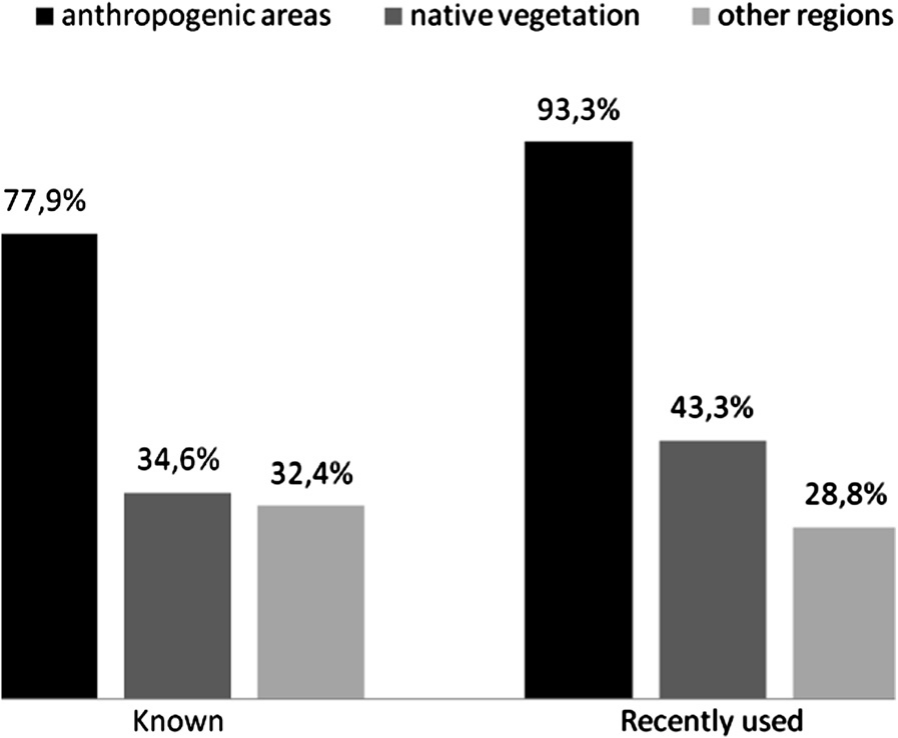
Percentage of ethnospecies with at least one citation for each collection site in the free lists (known) and lists of recently used plants in Horizonte community, NE Brazil.

The total values in Table 2 indicate the number of ethnospecies with at least one citation for each collection site; these values are expressed in percentages in Figure 2.

The importance of anthropogenic areas as sites for collecting medicinal plants is even more apparent when analyzing the plants that were recently used (Figure 2). This information about the collection site, although recorded using interviews, corroborates the results obtained by Gavin [8], who directly recorded the collection sites of plants used in the Peruvian Amazon. He reported more collection of medicinal plants in secondary vegetation that had developed after cultivated land was abandoned than in primary forest. Similar percentages of plants collected in anthropogenic areas (between 74% and 80%) were obtained by Frei *et al.*[36] in their study with the Mixe and Zapotec indigenous groups in Mexico.

The percentage of medicinal plants from other regions was lower in the group of recently used plants compared with the known plants (Figure 2). This finding may be an effect of the accessibility of these resources because the plants from other regions must be purchased commercially (involving monetary costs) or acquired during travel to surrounding Caatinga sites. In contrast, the percentage of native vegetation plants was higher among the plants recently used than for the known plants, indicating that the native vegetation was more important in terms of the practical level or actual use of medicinal plants.

### Cognitive aspects of use

Using the information from the free lists, a strong correlation between salience and use value was found (rs = 0.950; p < 0.001), i.e., plants with a high use potential are listed by more people and are ranked higher within the lists, or in other words, people quickly remember plants with a high use potential or versatility.

Herbs and trees exhibited no significant differences in use values (H = 0.0111, p > 0.05), and these groups did not differ significantly from shrubs (H = 3.3941 and H = 2.4536, respectively, p > 0.05). Herbs, trees, and shrubs had use values significantly different from that of climbers (H = 7.0096, H = 6.3171, and H = 3.9326, respectively, p < 0.05). Trees had, on average, higher use values (0.205 ± 0.283), followed by herbs (0.201 ± 0.267), shrubs (0.098 ± 0.143), and climbers (0.042 ± 0.054). However, the use value for plants is distributed independently from lignification because the use values for woody plants did not significantly differ from those of non-woody plants (H = 0.5186 p > 0.05).

According to the apparency hypothesis based on biochemical fundamentals, non-woody plants and herbs were expected to have higher use values and therefore higher versatility, but the results indicate that medicinal trees, shrubs, and herbs are equally versatile, and woody plants are as versatile as non-woody ones.

Among the native medicinal plants used by the rural communities that inhabit Caatinga areas, the trees and shrubs exhibited higher occurrences of quantitative compounds (phenols and tannins) and qualitative compounds (alkaloids, triterpenes, and quinones) [26]. These results partially meet the predictions made based on the biochemical fundamentals of the apparency hypothesis, which may, for example, help explain the versatility of apparent plants in the studied pharmacopoeia. In the same study, Almeida *et al*. [26] found that medicinal trees and shrubs were the habits with higher relative importance, above herbs, which are similar results to those obtained in the present study, indicating the possibility of a similarity in this respect between the Cerrado and Caatinga pharmacopoeias. Contrasting results were obtained in rainforests such as the Bolivian Amazon, where herbs have, on average, a higher medicinal use value than all the other habits (trees, shrubs, and climbers) [30].

Use value was also not influenced by origin; there were no differences between native and exotic plants (H = 3.2457 p > 0.05), indicating that the Horizonte residents have exploited as many medicinal uses for exotic plants as for native medicinal species. According to the diversification hypothesis, this result may indicate that exotic plants have been included within the pharmacopeia to treat specific diseases because they contain biochemical compounds that do not exist in the local pharmacopoeia or have higher concentrations of those already present [37]. Cultural and biochemical analyses would have to be conducted to delve into the cognitive aspects of the use of exotic plants within this pharmacopoeia.

### Ethnospecies habit and commercial value

Nine medicinal ethnospecies, all of which were native, were associated with more than 75% affirmative citations for commercial value; of these ethnospecies, 8 were woody (5 trees and 3 shrubs), and one was a non-woody (herb) plant. Again separating the native vegetation outside and within the FLONA, it was found that for five of the ethnospecies, 75% or more of the citations were for collection within the FLONA, three were exclusively from anthropogenic areas (100% of the citations were for this collection site), and one had less than 75% of its citations for any particular collection site (Table 3).

**Table 3:** Commercially important medicinal ethnospecies with more than two informants and 75% or more citations affirmative for commercial value (collection sites expressed as the percentage of citations for that site, and commercial value expressed as the percentage of citations of affirmative commercial value of the plant)

**Ethnospecies/species**	**Habit**	**Collection sites**	**Part sold**	**Commercial value**	**No. of informants**
”Fava d”anta’ tree/*Dimorphandra gardneriana Tul.*	Tree	52.7% FLONA, 18.2% nat. veg. outside FLONA, 29.1% anthropogenic area	Fruit	100%	29
Malva/*Waltheria indica* L.	Herb	100% anthropogenic area	Inflorescence	100%	2
‘Pequi’/*Caryocar coriaceum Wittm.*	Tree	76.3% FLONA, 5.3% native vegetation outside FLONA, 18.4% anthropogenic area	*Oil from fruit and seed	98.28%	58
‘Janaguba’/*Himatanthus drasticus* (Mart.) Plumel.	Tree	85.6% FLONA, 11.0% native vegetation outside FLONA, 3.4% anthropogenic area	Latex, stem bark	88.89%	99
‘Catuaba’/*Erythroxylum ampliofolium* (Mart.) O.E. Schulz	Shrub	81.8% FLONA, 12.1% native vegetation outside FLONA, 3.0% anthropogenic area	Stem bark	80%	30
‘Barbatimão’/*Stryphnodendron rotundifolium* Mart..	Tree	90.7% FLONA, 7.6% native vegetation outside FLONA, 1.7%. anthropogenic area	Stem bark	75.70%	107
Cassava/*Manihot esculenta* Crantz	Shrub	100% anthropogenic area	*Gum	75%	4
‘Mangaba’/*Hancornia speciosa* Gomes	Tree	87.5% FLONA, 9.4% native vegetation outside FLONA, 3.1% anthropogenic area	Latex, stem bark	75%	56
‘Urucum’/*Bixa orellana* L.	Shrub	100% anth anthropogenic area	Seed	75%	8

Horizonte community, NE Brazil.

*product obtained from any part of the plant.

It was expected that non-apparent plants, non-woody plants or plants with an herbaceous habit, characterized by having highly bioactive compounds, would have more ethnospecies with high percentages of affirmative citations for commercial value. However, contrary to expectations, the woody trees had a higher number of commercially important ethnospecies. Of the 12 plant species most commonly sold in the markets in the city of Belem in the Brazilian Amazon, nine are woody natives (six trees, two shrubs, and one climber) and the remaining three are herbaceous exotic plants (extracontinental origin) [38]. In the Caatinga region, medicinal plant use is usually associated with removing the bark from tree species [17], and the bark from native trees is usually sold in popular markets [39]. These studies indicate that in other ecosystems, trees also constitute the habit of greater economic importance and are mostly native species, such as the trees shown in Table 3. The economic importance of native trees in different ecosystems may be due to cultural factors, such as the perception that naturally growing plants have more healing power [36], and the biochemical diversity of these plants, as in the case of Caatinga [26]. These factors together with resource availability would lead native trees, dominant in the local vegetation, to be an excellent target for commercialization.

Within the group of commercially important plants, five trees and one shrub were strongly associated with collection in the FLONA, the conservation unit having the role of supplying commercially valuable medicinal plants to the Horizonte residents. Relatively high percentages of collection in anthropogenic areas for some of these trees could indicate some type of species management, as in the case of the ’fava d’anta’ tree (*Dimorphandra gardneriana Tul.*) and ‘pequi’ (*Caryocar coriaceum* Wittm.) (Table 3), which are species that provide substantial income for the Horizonte residents. The collection of ‘barbatimão’ (*Stryphnodendron rotundifolium* Mart.), ‘janaguba’ (*Himatanthus drasticus* (Mart.) Plumel.), ‘mangaba’ (*Hancornia speciosa*), and ‘catuaba’ (*Erythroxylum ampliofolium* (Mart.) OE Schulz) is prohibited by IBAMA (Instituto Brasileiro do Meio Ambiente e dos Recursos Naturais Renováveis [Brazilian Institute of Environment and Renewable Natural Resources]). Based on the results obtained, one can predict that the collection of these at the commercial level is performed within the FLONA, and thus, these species must be a conservation priority, for which the development of a management plan is recommended.

The commercially valuable parts of cassava (*Manihot esculenta* Crantz) and ‘urucum’ (*Bixa orellana* L.) (Table 3) are also popularly consumed as food, diffusing their medicinal commercial value. Likewise, the commercial value of the two types of oil from ‘pequi’ is reinforced by their use as food [40]. The ‘fava d’anta’ tree is considered medicinal by the Horizonte residents primarily because the Merck pharmaceutical industry purchases considerable amounts each year to use in medicines [18]. The uhaloa (*Waltheria indica* L.), also bought by Merck, was cited by only two informants, indicating that this plant has little commercial importance for the community.

There was no correlation between the commercial values of the plants and their use values (rs = -0.193, p > 0.05), a correlation that was also non-significant (rs = -0.032, p > 0.05) for the native pharmacopoeia. This correlation, negative in both cases, indicates that there are plants with high use values and few citations for commercial values (see Table 1), suggesting that the economic importance of medicinal plants, regardless of their versatility, can be related to other factors, i.e., the medicinal effectiveness for which the plant is indicated can result in a high commercial demand, or the low incidence of the disease they treat would result in lower commercial demand.

## Conclusions

From the perspective of its biochemical fundamentals, the apparency hypothesis does not have predictive potential to explain the use value and commercial value of medicinal plants. In other hand, the herbaceous habit showed the highest ethnospecies richness in the community pharmacopeia, which is an expected prediction, corroborating the apparency hypothesis.

Exotic plants have a strong influence on this pharmacopoeia because these plants are determinants of the greater prominence of the herbaceous habit and its practical value as well as the importance of anthropogenic areas. Moreover, the exotic plants are as versatile as native plants in treating diseases.

The FLONA has demonstrated its role as a source of commercially valuable medicinal plants for the Horizonte residents. Moreover, conservation units such as the FLONA are considered to play an important role in conserving the local knowledge of native medicinal plants because excluding exotic plants showed that the native pharmacopoeia is mostly woody and from native vegetation sites.

The results found here, in agreement with those obtained by other authors, indicate that within a pharmacopeia, there are ecological, biochemical, and cultural variables that interact and determine the use and importance of plants in different cognitive and applied aspects. More studies in different ecosystems that include environmental and cultural variables are necessary to increase our understanding of the behavior of tropical pharmacopoeias in relation to the apparency hypothesis.

## Competing interests

The authors declare that they have no competing interests.

## Authors’ contributions

AL was the main author responsible for conducting the study, obtaining and analyzing data, and writing the manuscript. Collection of the plant specimens was also carried out by AL, and UPA assisted with identifications. UPA, MFTM and ELA contributed to the design of the study, interpretation of the findings and preparation of the manuscript. All authors read and approved the final manuscript.

## References

[B1] Feeny P Wallace JW, Nansel RL Plant Apparency and chemical defense Biological Interactions between Plants and Insects. Recent Advances in Phytochemistry. Volume 10 1976 New York: Plenum Press 1 40

[B2] Rhoades DF Cates RG Wallace JW, Nansel RL Toward a general theory of plant antiherbivore chemistry Biological Interactions between Plants and Insects. Recent Advances in Phytochemistry. Volume 10 1976 New York: Plenum Press 169 213

[B3] Albuquerque UP Lucena RFP Can apparency afect the use of plants by local people in tropical forest? Interciencia 2005 30 8 506 511

[B4] Lucena RFP Araújo EL Albuquerque UP Does the local availability of woody Caatinga plants (northeastern Brazil) explain their use value? Econ bot 2007 61 4 347 361 10.1663/0013-0001(2007)61[347:DTLAOW]2.0.CO;2

[B5] Lucena RFP Medeiros PM Araújo EL Alves AGC Albuquerque UP The ecological apparency hypothesis and the importance of useful plants in rural communities from northeastern Brazil: an assessment based on use value J Enviro Manage 2012 96 106 115 10.1016/j.jenvman.2011.09.001 22208403

[B6] Albuqueque UP Ramos MA Melo JG New strategies for drug discovery in tropical forests based on ethnobotanical and chemical ecological studies J Ethnopharmacol 2012 140 197 201 10.1016/j.jep.2011.12.042 22226976

[B7] Chazdon RL Coe FG Ethnobotany of woody species in second-growth, old-growth, and selectively logged forests of northeastern Costa Rica Conserv Biol 1999 13 6 1312 1322 10.1046/j.1523-1739.1999.98352.x

[B8] Gavin MC Conservation implications of rainforest use patterns: mature forests provide more resources but secondary forests supply more medicine J Appl Ecol 2009 46 1275 1282

[B9] Thomas E Van Damme P Plant use and management in homegardens and swiddens: evidence from the Bolivian Amazon Agrofor Syst 2010 80 131 152 10.1007/s10457-010-9315-x

[B10] Bennett BC Prance GT Introduced plants in the indigenus farmacopedia of northern South America Econ bot 2000 54 1 90 102 10.1007/BF02866603

[B11] Stepp JR Moerman DE The importance of weeds in ethnopharmacology J Ethnopharmacol 2001 75 19 23 10.1016/S0378-8741(00)00385-8 11282438

[B12] Voeks RA Tropical forest healers and habitat preference Econ bot 1996 50 4 381 400 10.1007/BF02866520

[B13] Voeks RA Disturbance pharmacopoeias: medicine and myth from the humid tropics Ann Assoc Am Geogr 2004 94 4 868 888

[B14] Phillips O Gentry AH The useful plants of Tambopata, Peru: I. Statistical hypothesis test with a new quantitative technique Econ bot 1993 47 1 15 32 10.1007/BF02862203

[B15] Albuquerque UP Re-examining hypotheses concerning the use and knowledge of medicinal plants: a study in the Caatinga vegetation of NE Brazil J Ethnobiol Ethnomed 2006 2 30 10.1186/1746-4269-2-30 16872499 PMC1557484

[B16] La Torre-Cuadros MLA Islebe GA Traditional ecological knowledge and use of vegetation in southeastern Mexico: a case study from Solferino, Quintana Roo Biodive Conserv 2003 2 2455 2476

[B17] Albuquerque UP Oliveira RF Is the use-impact on native Caatinga species in Brazil reduced by species richness of medicinal plants? J Ethnopharmacol 2007 113 156 170 10.1016/j.jep.2007.05.025 17616289

[B18] IBAMA Plano de Manejo da Floresta Nacional do Araripe 2004 Brasília: Instituto Brasileiro do Meio Ambiente e dos Recursos Naturais Renováveis

[B19] Albuquerque UP Lucena RFP Alencar NL Albuquerque UP, Lucena RFP, Cunha L, Alves RRN Methods and techniques used to collect ethnobiological data Methods and Techniques in Ethnobiology and Ethnoecology 2014 New York: Springer

[B20] Begon M Townsend CR Harper JL Ecology: From Individuals to Ecosystems 2006 4 Oxford: Blackwell Publishing Ltd

[B21] Costa IR Araújo FS Organização comunitária de um encrave de Cerrado sensu stricto no bioma Caatinga, chapada do Araripe, Barbalha, Ceará Acta Bot Bras 2007 21 2 281 291 10.1590/S0102-33062007000200004

[B22] Rossato SC Leitão-Filho HF Begossi A Ethnobotany of Caiçaras of the Atlantic Forest coast (Brazil) Econ bot 1999 53 387 395 10.1007/BF02866716

[B23] Borgatti SP Natick MA Anthropac 4.0 1996 Natick: Analytic Technologies

[B24] Ayres M Ayres JM Ayres DL Santos AA BioEstat: Aplicações Estatísticas nas Áreas das Ciências Biológicas e Médicas 2007 Belém: Sociedade Civil Mamirauá: MCT-CNPq

[B25] Albuquerque UP Medeiros PM Almeida ALS Monteiro JM Lins-Neto EMF Melo JG Santos JP Medicinal plants of the Caatinga (semi-arid) vegetation of NE Brazil: a quantitative approach J Ethnopharmacol 2007 114 325 354 10.1016/j.jep.2007.08.017 17900836

[B26] Almeida CFCBR Silva TC L e Amorim ELC De S Maia MB Albuquerque UP Life strategy and chemical composition as predictors of the selection of medicinal plants from the Caatinga (northeast Brazil) J Arid Environ 2005 62 127 142 10.1016/j.jaridenv.2004.09.020

[B27] Phillips O Gentry AH The useful plants of Tambopata, Peru: II. Additional hypothesis testing in quantitative ethnobotany Econ bot 1993 47 1 33 43 10.1007/BF02862204

[B28] Caniago I Siebert ST Medicinal plant ecology knowledge and conservation in Kalimantan Indonesia Econ bot 1998 52 3 229 250 10.1007/BF02862141

[B29] Gavin MC Changes in forest use value through ecological succession and their implications for land management in the Peruvian Amazon Conserv Biol 2004 18 6 1562 1570 10.1111/j.1523-1739.2004.00241.x

[B30] Thomas E Vandebroek I Van Damme P Valuation of forests and plant species in indigenous territory and national park Isiboro-Sécure, Bolivia Econ bot 2009 63 3 229 241 10.1007/s12231-009-9084-5

[B31] Monteiro JM Albuquerque UP Lins-Neto EMF Araújo EL Amorim ELC Use patterns and knowledge of medicinal species among two rural communities in brazil’s semi-arid northeastern region J Ethnopharmacol 2006 105 173 186 10.1016/j.jep.2005.10.016 16298502

[B32] Medeiros MFT Albuquerque UP The pharmacy of the Benedictine monks: the use of medicinal plants in northeast Brazil during the nineteenth century (1823–1829) J Ethnopharmacol 2012 139 280 286 10.1016/j.jep.2011.11.014 22115750

[B33] Marques VRB Natureza em boiões: medicinas e boticários no Brasil setecentista 1999 Campinas: Editora da Unicamp

[B34] Baider C Tabarelli M Mantovani W O banco de sementes de um trecho de floresta atlântica montana (São Paulo, Brasil) Rev Bras Biol 1999 59 2 319 328 10.1590/S0034-71081999000200014

[B35] Ratter JA Ribeiro JF Bridgewater S The brazilian Cerrado vegetation and threats to its biodiversity Ann Bot 1997 80 223 230 10.1006/anbo.1997.0469

[B36] Frei B Sticher O Heinrich M Zapotec and mixe use of tropical habitats for securing medicinal plants in Mexico Econ bot 2000 54 1 73 81 10.1007/BF02866601

[B37] Alencar NL De Sousa Araújo TA Cavalcanti De Amorim EL Albuquerque UP The inclusion and selection of medicinal plants in traditional pharmacopoeias—evidence in support of the diversification Econ bot 2010 64 1 68 79 10.1007/s12231-009-9104-5

[B38] Shanley P Luz L The impacts of forest degradation on medicinal plant use and implications for health care in eastern Amazonia Bioscience 2003 53 6 573 584 10.1641/0006-3568(2003)053[0573:TIOFDO]2.0.CO;2

[B39] Monteiro JM Araújo EL Amorim ELC Albuquerque UP Local markets and the commerce of medicinal plants: a review with emphasis in Brazil Econ bot 2010 64 4 352 366 10.1007/s12231-010-9132-1

[B40] Gonçalves CU Os piquizeiros da Chapada do Araripe, 25(1) Revista de Geografia 2008 Recife: UFPE – DCG/NAPA 88 103

